# Listening to 15 Hz Binaural Beats Enhances the Connectivity of Functional Brain Networks in the Mental Fatigue State—An EEG Study

**DOI:** 10.3390/brainsci12091161

**Published:** 2022-08-30

**Authors:** Xinlu Wang, Hongliang Lu, Yang He, Kewei Sun, Tingwei Feng, Xia Zhu

**Affiliations:** Department of Military Medical Psychology, Air Force Medical University, Xi’an 710032, China

**Keywords:** mental fatigue, binaural beats, relaxing music, EEG

## Abstract

Introduction: It is clear that mental fatigue can have many negative impacts on individuals, such as impairing cognitive function or affecting performance. The aim of this study was to investigate the role of sound interventions in combating mental fatigue. Method: The subjects were assessed on various scales, a psychomotor vigilance task (PVT) task, and a 3 min resting-state electroencephalogram (EEG), followed by a 20 min mental fatigue–inducing task (Time Load Dual Back, TloadDback), during which subjects in different condition groups listened to either 15 Hz binaural beats, 40 Hz binaural beats, relaxing music, or a 240 Hz pure tone. After the mental fatigue–inducing task, subjects were again assessed on various scales, a PVT task, and a 3 min resting-state EEG. Results: After the fatigue-inducing task, there was no significant difference between the four groups on the scales or the PVT task performance. In TloadDback, the accuracy rate of the 40 Hz binaural beats group and the relaxing music group decreased in the middle stage of the task, while the 15 Hz binaural beats group and the 240 Hz pure tone group remained unchanged in all stages of the task. The EEG results showed that after fatigue inducement, the average path length of the 15 Hz binaural beats group decreased, and local efficiency showed an increasing tendency, indicating enhanced brain network connectivity. Meanwhile, the 240 Hz pure tone group showed enhanced functional connectivity, suggesting a state of mental fatigue in the group. Conclusions: The results of this study show that listening to 15 Hz binaural beats is a proven intervention for mental fatigue that can contribute to maintaining working memory function, enhancing brain topological structure, and alleviating the decline in brain function that occurs in a mentally fatigued state. As such, these results are of great scientific and practical value.

## 1. Introduction

Modern life, with its high-stress life and work, has resulted in mental fatigue becoming a common phenomenon, including feelings of exhaustion, depletion, an aversion to continuing tasks, and a decrease in commitment to ongoing tasks [1,2]. Mental fatigue can negatively affect individual-level cognitive abilities, emotional management, and behavioral control [3,4], causing decreased alertness [5], a narrowed attention span [6,7], diminished short-term memory [8], and reduced emotion regulation [3], leading to increased risky decision-making [9] and decreased work efficiency. Fatigue also leads to accidents in different fields, such as in the medical industry [10], on the military field [11,12], and in transportation [11,13,14].

It is necessary to eliminate the negative effects of mental fatigue with the help of intervention techniques, and it has been shown that naps [15,16,17,18,19], rest [20], and caffeine consumption [21,22] are common interventions.

Recently, researchers have proposed sound intervention [23,24,25], olfactory intervention [26], and other mental fatigue countermeasures, among which sound interventions have gained much attention. As a non-invasive form of fatigue intervention, sound interventions include music interventions and binaural beats interventions. Sound is a regular vibration with an emotional arousal effect [27,28], which has been shown to enhance alertness [29,30], reduce stress [31], treat post-traumatic stress disorder [32,33], reduce mental fatigue [34], and is an effective method used against driving drowsiness and fatigue [35,36]. Music affects the arousal level and emotional state of listeners. It has been evidenced that positive emotions can enhance cognitive flexibility [23] and have a beneficial effect on cognitive performance [37], as well as being an intervention for mental fatigue [38]. Neuroimaging studies have shown that listening to favorite music increases the frontal functional connectivity of subjects [23]; motivational music has an ergogenic effect on physical experience and cognitive performance by increasing arousal levels and enhancing emotional experiences [39], while the smoothing effect of listening to relaxing music [40] helps to reduce mental fatigue in listeners [35]. Music not only has a positive impact on people, but also has a certain negative impact on some individuals, due to factors such as musical attributes [41], situational attributes, and individual differences [42]. For example, different types of music induce different emotions; listening to rap genres was found to generate sadness [43], listening to loud music will reduce driving performance and increase violations such as speeding [41,44], while fast-paced music increases heart rate [41]. Alikonis et al. found that music may divert cognitive resources, distract attention, and reduce performance on tasks that require focused attention [45]. In other words, there is no consensus on the role of music in counteracting mental fatigue. Interventions based on the electroencephalogram (EEG) spectrum have been used in the diagnosis and treatment of people with various symptoms. Neurofeedback technology based on EEG spectrum analysis has had a positive therapeutic effect in the treatment of insomnia, anxiety, ADHD, and other diseases [46]. After 15 alpha training sessions of 30 min each, people with generalized anxiety disorder had an increase in alpha and theta wave amplitude in the occipital area, an improvement in global functioning level, and fewer symptoms [47]. Not only effective in people with symptoms, studies have shown that EEG spectrum intervention can also have an impact on cognitive function in healthy people. Binaural beats intervention is a sound intervention that can have similar effects to EEG spectrum intervention. Binaural beats are an auditory illusion in which individuals can perceive a third tone at a frequency equal to the difference between two separate tones when two sounds of slightly different frequencies are presented to each ear [48]. For example, if a 260 Hz tone is presented in the right ear and a 240 Hz tone in the left ear, a 20 Hz tone is perceived in the brain. Binaural beats have a modulatory effect on cognitive function [49,50]. When two ears receive two pure tones of different frequencies, the stimuli are transmitted along the auditory pathway, then phase-sensitive neurons in the hypothalamus are stimulated and the beats are recognized in the superior olivary complex [51,52], at which time, the frontal and parietal lobes associated with cognitive function are activated and cognitive ability, such as attention and verbal memory, is enhanced [53,54]. High-frequency beats are associated with alertness and attentional focus [55]. Beauchene et al. found that subjects receiving 15 Hz binaural beats had significant activation of the frequency band when performing the N-Back task, producing a brain network with higher information transfer characteristics [53], whereas binaural beats in the gamma band at 40 Hz enhanced attentional focus and boosted alertness and attention [56]. However, there is disagreement among existing studies regarding the effects of binaural beats on brain activity and cognitive performance. Although some studies have shown that auditory beat stimulation (ABS) plays a role in reducing anxiety [57], enhancing emotional states, and even modulating verbal memory capacity [58], other studies have not observed effects on emotion evocation [59], vigilance maintenance [60], or any changes in brain mechanisms [59]. To verify whether binaural beats and relaxing music have an intervention effect on the state of mental fatigue, we plan to use 15 Hz binaural beats, 40 Hz binaural beats, and relaxing music in our study as interventions to explore their impact and to provide new evidence for the effect of at least one of these interventions based on prior inconsistent, even contradictory, results. 

Brain connectivity analysis examines how units within the nervous system are connected, including the functional connectivity patterns of causal interactions between different individual neurons, neuronal populations, or brain regions. Functional connectivity refers to the statistical dependence between spatially separated neuronal events, revealing relationships between similar activation patterns in different brain regions [53]. In the awake state, brain connectivity is good and information transfer is efficient, in the mental fatigue state, however, information exchange between brain regions is elevated and functional connectivity is enhanced to maintain normal brain function. Based on graph theory, features of brain networks can be calculated to analyze brain network characteristics, such as average path length, clustering coefficients, etc. When maintaining a better mental state, the connectivity network obeys the principle of small-world organization with shorter path lengths and higher clustering coefficients; however, when in a fatigued state, average path lengths become longer and clustering coefficients become lower. The present study recorded EEG signals and analyzed inter- and intra-neuronal interactions, relationships, and characteristic changes represented by electrode locations using connectivity analysis and graph theoretical methods, which facilitated further exploration of the effects produced by different sound interventions on the cerebral cortex.

To the best of our knowledge, few existing studies have directly investigated the effects of sound intervention on mental fatigue. Most experiments have conducted sound intervention after mental fatigue inducement to investigate the intervention effects and neural mechanisms, while few have conducted the intervention simultaneously with fatigue inducement. In this study, we proposed to present binaural beats at different frequencies, relaxing music, or pure tone simultaneously with mental fatigue inducement, record changes in subjective scale scores and behavioral task performance before and after mental fatigue inducement to explore the intervention effects, and use EEG techniques to explore the changes in brain function. We hypothesized that after the induction of mental fatigue, the control group (the group that received pure tone) had higher Visual Analogue Scale-Fatigue (VAS-f) scores and Brunel Mood Scale (BRUMS) scores, a prolonged mean reaction time on the PVT task, and decreased accuracy on TloadDback compared with the intervention group. Meanwhile, functional connectivity was enhanced to maintain the normal working state under the fatigued state in the control group. In the intervention group, at least one group showed a trend opposite to that of the control group, indicating that this method is an effective intervention for combating mental fatigue.

## 2. Materials and Methods

### 2.1. Participants

**Figure 1:**
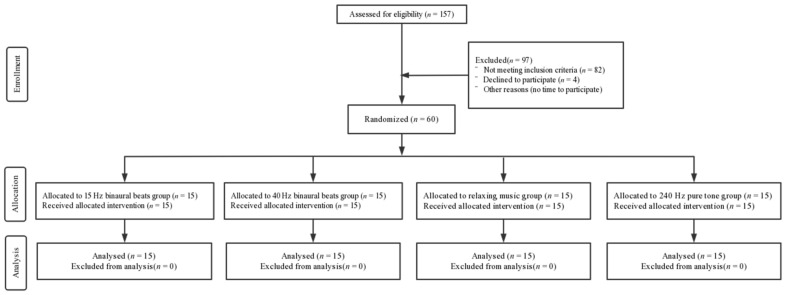
CONSORT flow diagram.

A total of 60 right-handed male subjects with visual acuity or corrected visual acuity of 1.0 and without any history of neurological or psychiatric disorders or substance abuse were recruited. They were randomly assigned to four groups by means of a random number: (1) 15 Hz binaural beats group (intervention); (2) 40 Hz binaural beats group (intervention); (3) relaxing music group (intervention); and (4) 240 Hz pure tone group (control). All subjects had normal sleep quality in the previous month (Pittsburgh Sleep Quality Index (PSQI) score < 16 and Epworth Sleepiness Scale (ESS) score < 10) and the Morningness–Eveningness Scale (MES) scores were moderate to intermediate (31 < scores < 69). The basic information regarding the subjects is shown in Table 1 and a CONSORT flow diagram is shown in Figure 1. All subjects were asked not to consume excitatory beverages or food such as coffee, alcohol, or tea 24 h before the experiment. All subjects volunteered to participate in the experiment and completed a written informed consent form. This study was conducted according to the Declaration of Helsinki and was approved by the Ethics Committee of Tangdu Hospital (2014-03-03), and has been registered with ClinicalTrials.gov (NCT02420470, http://www.clinicaltrials.gov/, accessed on 1 March 2022).

### 2.2. Material

#### 2.2.1. Mental Fatigue-Inducing Task: The Improved Version of TloadDback (Time Load Dual Back)

The improved TloadDback is an adaptive mental fatigue–inducing task completed in two days, with the participant’s best processing capability determined on the first day and formal fatigue inducement performed on the second day. 

The procedures on the first day are divided into phase 1 and phase 2. Phase 1 includes the odd/even decision task and the 1-back task. The odd/even decision task is performed first. Participants must judge whether a number displayed on a screen is odd or even. If the number is odd, they should press “1” on a numeric keypad with their right hand, or press “2” if the number is even. When the accuracy rate for this task reaches 85%, then the odd/even decision task is finished; if the accuracy rate is not achieved, the task is repeated for another block until 85% is reached. In the 1-back task, participants have to judge whether a letter is the same as the previous one. When the same, the “Z” key is pressed with the left hand; if different, the “X” key is pressed. When the accuracy rate for this block reaches 85%, the subject enters phase 2; otherwise, another block of the 1-back task is repeated until the accuracy rate is achieved.

In phase 2, participants carry out a dual task combining the odd/even decision task and the 1-back task. In this phase, stimuli are presented in the sequence of “digit-letter-digit-letter” with a stimulus time duration of 1500 ms. The accuracy rate in this phase is calculated using a weighted calculation method in which the accuracy rate for the odd/even decision task accounts for 35%, and the accuracy rate for the 1-back task accounts for 65%. If the total accuracy rate does not reach 85%, another block with a duration of 1500 ms is performed until the minimum accuracy rate is met. When the total accuracy rate reaches 85%, a double task with a reduced presentation time of 1400 ms per stimulus is performed. When the accuracy rate meets 85% again, the duration in the next block is shortened by 100 ms overall. This step continues until the total accuracy rate falls below 85%. When it falls below 85% for the first time, two blocks are given with the same stimulus duration as the failed block; if the weighted accuracy rate in each of these two blocks is below 85%, the stimulus duration of that time plus 100 ms is recorded, which is the final stimulus duration used for day 2, and the procedure for day 1 is finished. However, if the accuracy rate in any of the two blocks is higher than 85%, the stimulus duration continues to decrease by 100 ms from this block until the final stimulus duration is obtained. On day 1, at the end of each block of phase 1 and phase 2, participants are reminded to rest and asked to complete the Visual Analogue Scale-Fatigue (VAS-f) to ensure that their mental state has not changed too much. 

**Figure 2:**
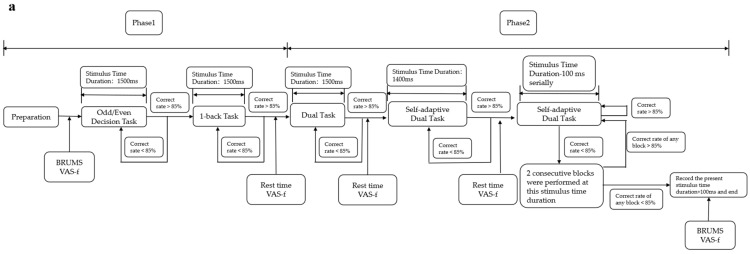

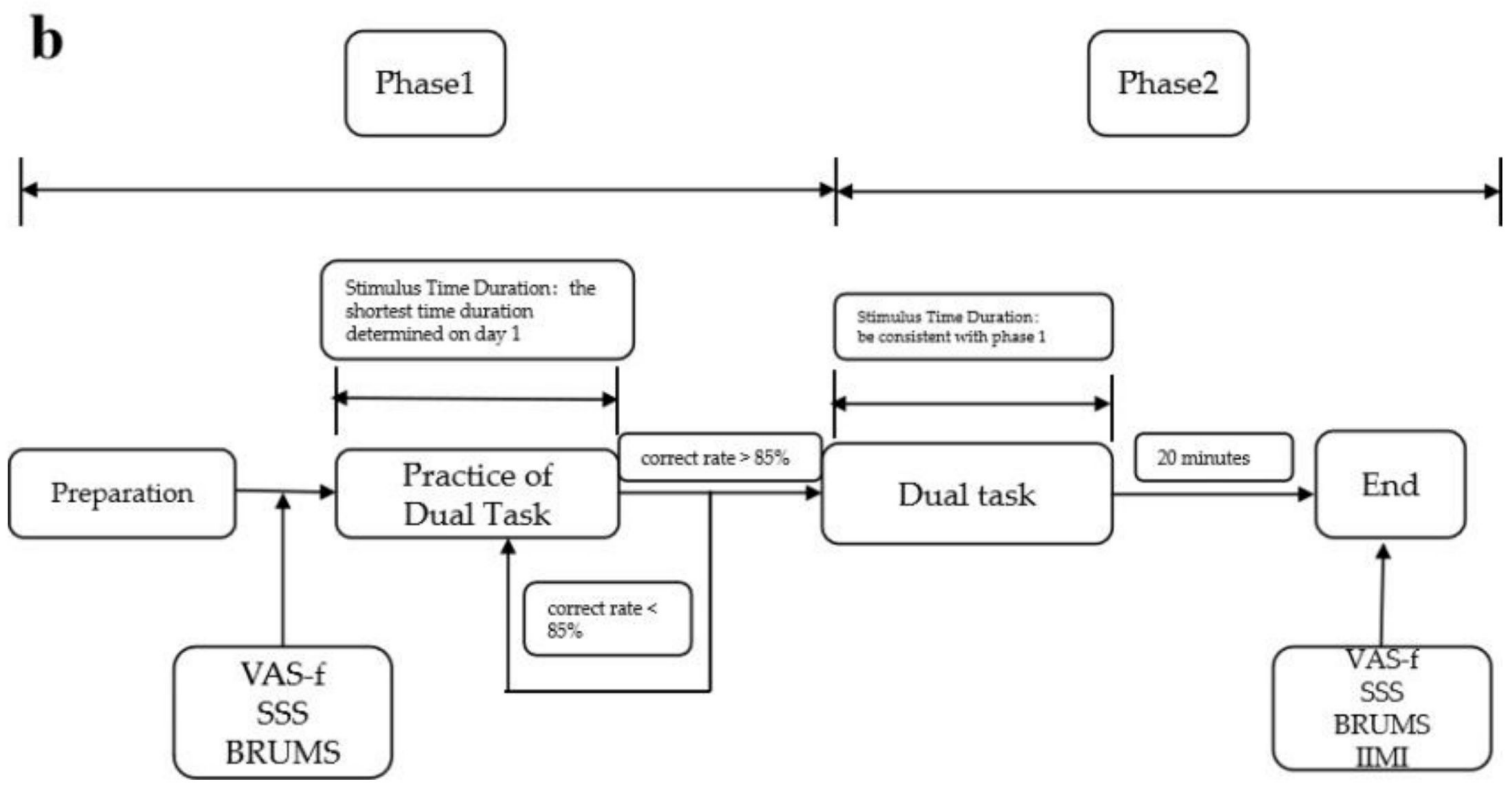
Flowchart of the improved version of the TloadDback task: (**a**) flowchart of day 1; (**b**) flowchart of day 2.

The procedure on day 2 also consists of two phases. Phase 1 is a practice phase in which a one-block dual task is presented using the fastest stimulus duration determined for each subject on day 1 to maintain 85% correctness. When 85% correctness is achieved, we believe that subjects are familiar again with the task and prepare for the formal experiment, then phase 2 is started. If it falls below 85%, another practice block is performed until the 85% correctness level is met. Phase 2 is the formal fatigue-inducing stage in which the stimuli are presented continuously for 20 min with no break. The specific procedures of the improved TloadDback task are shown in Figure 2. 

#### 2.2.2. Scales and Paradigms

##### Visual Analogue Scale-Fatigue (VAS-f)

The Visual Analogue Scale is a subjective measure that typically uses a straight line of a specific length (typically 100 mm) with extreme descriptions at each end. Individuals mark a point on that line whose distance from an extreme description represents the individual’s evaluation or attitude. To obtain the scores of fatigue quickly and efficiently, only one item was used in our study, which was “Not at all fatigued—Extremely fatigued”, rated on a scale of 0–100 [61,62].

##### Intrinsic Motivation Inventory (IMI)

The Intrinsic Motivation Inventory can be used to self-assess subjective feelings related to the target activity in an experiment and contains six subscales: interest/fun, perceived competence, effort, perceived stress and tension, value/usefulness, and perceived choice. In this study, the “effort” subscale was used to allow subjects to self-assess their effort while performing the fatigue-inducing task TloadDback.

##### Other Cognitive Scales and Tasks

This study used the Pittsburgh Sleep Quality Index (PSQI), the Epworth Sleepiness Scale (ESS), and the Morningness–Eveningness Scale (MES) as screening scales for subjects’ inclusion. Furthermore, the Visual Analogue Scale-fatigue, the Brunel Mood Scale (BRUMS), and the Psychomotor Vigilance Task (PVT) were used before and after mental fatigue inducement, and the “effort” subscale in the Intrinsic Motivation Scale was filled out only after fatigue inducement. We will not describe the above scales and paradigm in detail as they are commonly used.

#### 2.2.3. Auditory Stimulation

As the effectiveness of binaural beats in the 100–300 Hz range has been demonstrated, 240 Hz was chosen as the primary carrier frequency in our study, and the corresponding frequency band stimuli were also set based on this frequency. The experimental conditions included: (1) 15 Hz binaural beats (R: 240 Hz, L: 255 Hz); (2) 40 Hz binaural beats (R: 240 Hz, L: 280 Hz); and (3) relaxing music (*Blue Lagoon*, *Melody of Love*, *Mother Nature*, *The Purple Butterfly*). Control conditions included 240 Hz pure tones (R: 240 Hz, L: 240 Hz). The right and left ears were denoted by R and L, respectively. The 15 Hz binaural beats and 40 Hz binaural beats were used as beta and gamma band stimuli, respectively. All audio required in our experiment was created using Audacity software (http://www.audacityteam.org, accessed on 20 March 2022).

For the relaxing music selection, 16 subjects were recruited to rate the three dimensions of arousal, pleasantness, and dominance for 16 pure music tracks from the *Mist* album by Bandari (www.bandari.net, accessed on 10 March 2022) using the self-assessment manikin (SAM); in addition, they reported the familiarity and enjoyment of each track. We excluded music that did not meet the requirements of inter-rater reliability, selected four pure music tracks with high pleasantness, medium arousal, low familiarity, and high enjoyment, and used Audacity software for synthesis.

The sound stimuli for each group were played through stereo headphones, and each subject set their own comfortable volume level before the start of the formal experiment as the volume to be received throughout TloadDback.

#### 2.2.4. Electroencephalogram (EEG)

The continuous electroencephalogram (EEG) was collected with the Brain Amplifier DC (Brain Products, München, Germany) using 32 Ag/AgCl electrodes mounted according to the extended international 10–20 system. The TP9 and TP10 electrodes were used as online references and the GND (ground channel) was used to eliminate power frequency interference. Data were sampled at 1000 Hz and a 250 Hz hardware low pass filter was applied. Electrode impedances were constantly kept below 5 kΩ.

#### 2.2.5. Experimental Procedure

A single-blind method was used in our experiment. To ensure that subjects were not influenced by extraneous factors such as practice effects, our study used a between-subjects design, with each subject completing the whole experiment over two days. Subjects with no excessive sleepiness, good sleep quality, and intermediate sleep habits in the previous month were screened using the Pittsburgh Sleep Quality Index, the Epworth Sleepiness Scale, and the Morningness–Eveningness Scale, and were randomly divided into four groups (15 Hz binaural beats group, 40 Hz binaural beats group, relaxing music group, and 240 Hz pure tone group).

**Figure 3:**
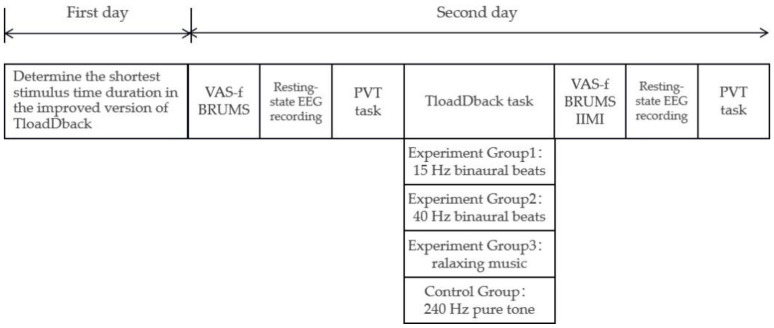
Protocol of formal experiment.

On the first day, we determined each subject’s individualized fastest processing speed in TloadDback; that is, the fastest stimulus time duration that each subject could reach while maintaining 85% correctness. On the second day, after preparation with the EEG, the subjects first completed the VAS-f, the BRUMS, underwent a 3 min resting-state EEG, then performed the PVT task, followed by a 20 min TloadDback, while subjects in different groups received a different sound intervention. Finally, they again underwent a 3 min resting-state EEG, performed the PVT task, and filled out the VAS-f, the BRUMS, and the “effort” subscale in the IMS. The full procedure took approximately 50 min (including preparation time) and is shown in Figure 3.

#### 2.2.6. Data Processing of Scales and Tasks

All statistical analyses were completed using SPSS Statistics, version 22 (IBM Corp, Armonk, NY, USA). All subjective and behavioral data were tested for normal distribution using the Shapiro–Wilk test and found to be normally distributed. For the subjective scales, scores were calculated for the VAS-f, BRUMS, and “effort” subscale in the Intrinsic Motivation Scale. For the PVT task, the mean reaction time, fastest (10th percentile) reaction time, and slowest (90th percentile) reaction time were calculated according to Lim’s standard [63]. The data derived from the 20 min TloadDback was divided by 4 min boundaries into 5 stages, and the overall trend was observed by comparing the 1-back task reaction time, the odd-even decision task reaction time, and the overall accuracy rate at each stage using repeated measures ANOVA. Furthermore, the total accuracy rate for each stage = the accuracy rate in the 1-back task × 0.65 + the accuracy rate in the odd-even decision task × 0.35.

#### 2.2.7. Data Processing of Electroencephalographic

EEG preprocessing was done using the open source toolbox EEGLAB [64] with MATLAB R2013b (The MathWorks Inc., Natick, MA, USA). The specific preprocessing steps included downsampling, re-referencing, filtering, bad channel removal, independent component analysis (ICA), and other artifact removals. The EEGLAB was used to analyze the connectivity of resting-state EEG before and after mental fatigue inducement for each group of subjects. Each electrode channel was defined as a network node, and the coherence values of three different frequency bands (theta, alpha, beta) were calculated to obtain a 30 × 30 connectivity matrix. Generally, there is no gold standard for choosing the threshold; we explored the whole threshold range from 0.4 to 0.85 with an interval of 0.05 and used the GRETNA toolbox to analyze the topological structure, including: (1) Clustering coefficient (Cp)—the clustering coefficient is a local feature of the network, representing the overlap of nodes that share connectivity between two adjacent nodes, and is used to assess the degree of integration of the network, reflecting the functional differentiation mechanism of the cerebral cortex, that is, the tightness of the connections between neurons in local brain functional areas. (2) Average path length (Lp)—the average path length is used to measure the transmission efficiency of the network. In complex networks, the smaller the average path length, the better the connectivity and efficiency of the network. (3) Nodal efficiency—the nodal efficiency of a given node characterizes the efficiency of the node in parallel information transmission in the network.

#### 2.2.8. Statistical Analysis

All original data are presented as mean ± SD and significance was taken as α= 0.05. The effect sizes for the repeated measures ANOVAs were calculated as partial eta squared (*η_p_*^2^). A 4 (condition: 15 Hz binaural beats, 40 Hz binaural beats, relaxing music, 240 Hz pure tone) × 2 (time points: pre-fatigue inducement, post-fatigue inducement) repeated measures ANOVA was used to analyze the scores of VAS-f, BRUMS, and PVT task performances, and was also used to compare the differences in the topological structure before and after mental fatigue inducement. Functional connectivity was FDR (false discovery rate) corrected for differences within brain regions (short-range connectivity) and between brain regions (long-range connectivity) and plotted. A 4 (conditions: 15 Hz binaural beats, 40 Hz binaural beats, relaxing music, 240 Hz pure tones) × 5 (time points: stage 1, stage 2, stage 3, stage 4, stage 5) repeated measures ANOVA was used to process the results of TloadDback. One-way repeated measures ANOVA was used to analyze the scores of the “effort” subscale in the Intrinsic Motivation Scale. 

## 3. Results 

### 3.1. Scales

#### 3.1.1. Visual Analogue Scale-Fatigue

**Figure 4:**
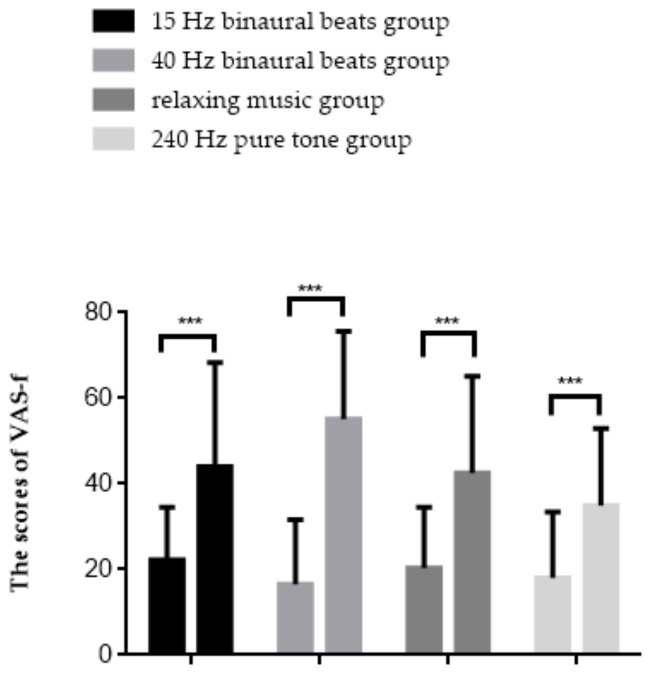
Results of the VAS-f. *** *p* < 0.001.

The results of the repeated measures ANOVA are shown in Figure 4, with a significant interaction effect between group and time point (*F*(3,56) = 4.798, *p* = 0.005, *η_p_*^2^ = 0.204). Simple effect analysis found that in all groups, the scores elevated after fatigue inducement (*p* < 0.001). The results of the VAS-f indicated that subjective fatigue scores were significantly increased in all groups of subjects after fatigue inducement, with the most obvious upward trend observed in the 40 Hz binaural beats group.

#### 3.1.2. BRUMS

A 4 × 2 repeated measures ANOVA for each dimension of the BRUMS scores revealed significant main effects on time for the dimension of anger, depression, tension, and energy (*F*(1,14) = 6.976, *p* = 0.011, *η_p_*^2^ = 0.111; *F*(1,14) = 2.251, *p* = 0.004, *η_p_*^2^ = 0.136; *F*(1,14) = 10.704, *p* = 0.002, *η_p_*^2^ = 0.160; *F*(1,14) = 28.801, *p* < 0.001, *η_p_*^2^ = 0.340); while main effects on group and time were both significant in the fatigue dimension (*F*(3,12) = 3.274, *p* = 0.028, *η_p_*^2^ = 0.149; *F*(1,14) = 69.186, *p* < 0.001, *η_p_*^2^ = 0.553). The results of the BRUMS indicated that scores on the anger dimension, depression dimension, tension dimension, and energy dimension were elevated after fatigue inducement regardless of the group; and scores on the fatigue dimension were significantly higher in each group after mental fatigue inducement, with no group and time point interaction.

#### 3.1.3. “Effort” Subscale in Intrinsic Motivation Scale

The “effort” subscale score was calculated for each group of subjects, and the results indicated that it did not differ significantly between groups (*F*(3,56) = 1.256, *p* = 0.298), demonstrating that subjects in different groups had the same level of subjective effort in completing the fatigue-inducing task TloadDback.

### 3.2. Behavioural Performances

#### 3.2.1. PVT

A 4 × 2 repeated measures ANOVA results showed that the main effects and the interaction effect in the mean reaction time, the fastest (10th percentile) reaction time, and the slowest (90th percentile) reaction time were not significant; that is, there was no change occurring in performances before and after fatigue inducement in the PVT task (all *p* > 0.05).

#### 3.2.2. TloadDback

**Figure 5:**
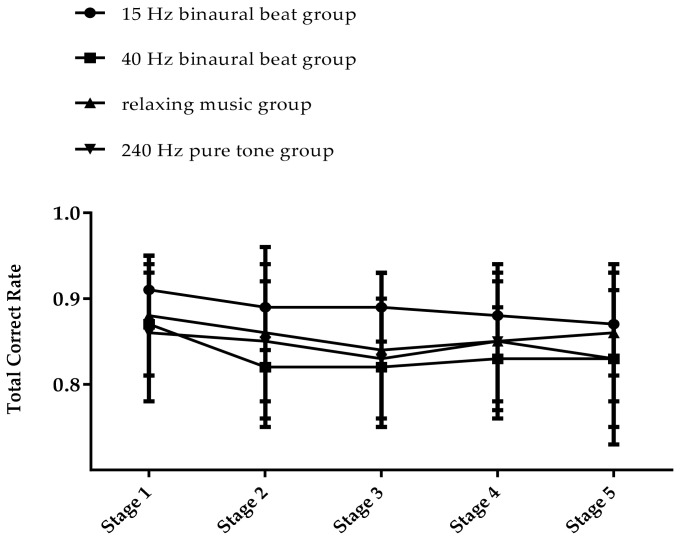
The trend in total accuracy rate of TloadDback (%).

We first calculated and compared the average cognitive processing speed confirmed on the first day of the TloadDback in each group, and the results of one-way ANOVA showed that there was no significant difference between the groups (F(3,56)= 2.037, *p* = 0.119). We then conducted a statistical analysis of the results of the second day of the TloadDback. A 4 × 5 repeated measures ANOVA was performed on the total accuracy rate and, from the results shown in Table 2, we can see that there is a significant main effect on time point (*F*(4,50) = 8.39, *p* < 0.001, *η_p_*^2^ = 0.402) and a significant interaction effect of group and time point (*F*(12,156) = 1.827, *p* = 0.048, *η_p_*^2^ = 0.123). Simple effect analysis revealed that the 40 Hz binaural beats group had a lower accuracy rate in stages 2, 3, and 4 than in stage 1 (*p* < 0.05), and the total accuracy rate in stage 5 was not significantly different from stage 1. The total accuracy rate in the relaxing music group had significant differences only in stage 1 and stage 3 (*p* < 0.01), and the accuracy rates of the 15 Hz binaural beats group and the 240 Hz pure tone group did not vary significantly in all five stages. The trends of the accuracy rates in each group are shown in Figure 5. In the TloadDback, the accuracy rates of the 15 Hz binaural beats group and the 240 Hz pure tone group in the five stages remained stable, while the 40 Hz binaural beats group and the relaxing music group had a reduced accuracy rate in the middle stage of the task, which returned to the initial level at the end stage of the task.

Repeated measures ANOVA for the 1-back task reaction time and the odd-even decision task reaction time revealed a significant main effect on time for the 1-back task reaction time (*F*(4,50) = 2.84, *p* = 0.034, *η_p_*^2^ = 0.185), and neither main effect nor interaction effect was significant on the odd-even decision task reaction time (*p* > 0.05). Furthermore, a post hoc test showed that there was no significant difference between the stages in each group for the 1-back task reaction time.

### 3.3. Electroencephalographic

#### 3.3.1. Functional Connectivity

**Figure 6:**
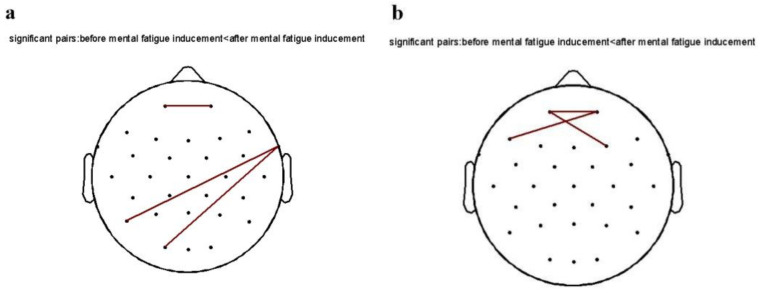
Diagram of the functional connection of the 240 Hz pure tone group in the eyes-opening state: (**a**) theta band; (**b**) alpha band.

As shown in Figure 6, the functional connectivity between the channels was plotted in MATLAB 2013b software using the power spectrum of each frequency band as an indicator, and FDR correction was made. After inducing mental fatigue, the functional connectivity of the 240 Hz pure tone group (FP1-Fp2; P7-FT10; O1-FT10) was elevated on the theta band in the eyes-opening state compared with that before fatigue inducement, while other groups had no significant changes. On the alpha band, functional connectivity (Fp1-Fp2; Fp2-F7; Fp1-F4) was elevated in the 240 Hz pure tone group compared with pre-fatigue inducement, with no significant changes in the remaining groups. Functional connectivity demonstrated that the 240 Hz pure tone group showed enhanced functional connectivity in the theta and alpha frequency bands after mental fatigue inducement, indicating a fatigued state after TloadDback.

#### 3.3.2. Topological Structure

The clustering coefficient (Cp), average path length (Lp), and nodal efficiency were calculated for each group before and after mental fatigue inducement, and the results are shown in Table 3. After mental fatigue inducement, in the theta band, the interaction effect on average path length was significant (*F*(3,56) = 3.239, *p* = 0.029, *η_p_*^2^ = 0.148) and decreased significantly in the 15 Hz binaural beats group (*p* < 0.05); the interaction effect on nodal efficiency was marginally significant (*F*(3,56) = 2.568, *p* = 0.063, *η_p_*^2^ = 0.121) and the 15 Hz binaural beats group showed an increasing trend (*p* < 0.05). The topological structure indicated higher functional efficiency and better connectivity in the brain of subjects in the 15 Hz binaural beats group after mental fatigue inducement.

## 4. Discussion

Among the existing mental fatigue intervention studies, there are few studies on sound intervention carried out at the same time as fatigue inducement. In this study, three different sound interventions were used as experimental groups, and a 240 Hz pure tone group was used as a control to investigate effective sound interventions.

In this study, the use of different sounds as mental fatigue intervention resulted in increased scores on VAS-f following fatigue inducement. Mega et al. manipulated motivation with rewards to control for mental fatigue, finding that the presence of rewards did not affect changes in the VAS-f scores [65], and Linnhoff et al. demonstrated that a single transcranial direct current stimulation (tDCS) ameliorated fatigue-induced physiological changes while not affecting subjective reports [66]. These studies both suggest that external manipulation has no significant effect on changes in subjective fatigue perception. In another study, the longer patients with multiple sclerosis performed a cognitive task, the higher the increase in reported cognitive fatigue, prompting the suggestion that their subjective feeling of fatigue seemed to depend on task length rather than on the actual feedback of their sensations [67]. Although this finding has not been confirmed in a population of healthy subjects, we speculate that the high sensitivity to elevated subjective fatigue feeling and low sensitivity to decline may be one reason for the lack of change in VAS-f scores after the intervention. The results of PVT suggested that vigilance before and after fatigue inducement did not change across groups. The PVT task is a simple vigilance task that is prone to ceiling effects, accounting for the results where subjects with different mental states show similar performances. This possibility was also demonstrated by the TloadDback task—because of the difficulty and cognitive load involved in that task, the scores of TloadDback showed different changes in the different groups. The performance of the 15 Hz binaural beats group remained stable, indicating that binaural beats intervention in the beta frequency band can alleviate the advanced cognitive function decline caused by mental fatigue. The scores in the 40 Hz binaural beats group and the relaxing music group decreased in the middle stage and improved finally in the last stage. We believe that there is both the emergence of mental fatigue and the recovery of mental resources in the middle stages. In the intermediate stage, the accuracy rate of TloadDback decreased as the subjects were in a state of mental fatigue, while at the same time, these stages of decreasing accuracy rate provided time for subjects to recover their mental resources, so that in the final stage, the subjects’ attention level temporarily increased and distraction decreased, maintaining a good task performance [20]. The performance of the 240 Hz pure tone group also remained stable, we speculate that because more brain areas were activated to maintain task performance in the fatigued state, both stable task performance and EEG indicators representing the fatigued state were present.

The scale is prone to inaccurate reporting due to subjective reasons, and the results of behavioral tasks are affected by subjects’ ability and task difficulty. Previous studies have shown that an EEG is an objective technical tool that can reflect the actual mental state of subjects [68]. Brain waves of different frequencies have different meanings and represent different mental states [69]. Theta frequency bands are related to intuition and automatic tasks, and often appear in a resting state. They are also the basic brain activity of learning and memory [70]. Alpha frequency bands are related to an individual’s arousal state and relaxation state [71]. When an individual closes his/her eyes and takes him/herself into a state of conscious relaxation, the alpha frequency band appears. In addition, it exists in perceptual, alertness, learning, and memory processes [72]. In the present experiment, after fatigue inducement, the 240 Hz pure tone group showed enhanced functional connectivity, with short-range connectivity within brain regions and long-range connectivity between brain regions in the theta and alpha frequency bands, indicating that this group is in a more alert learning and memory state. During the awake state, the brain requires just a few functional connections to maintain the required level of cognition; whereas in a fatigued state, more brain areas must be activated to work collaboratively, thereby increasing the synchronization needed to maintain the cognitive capacity required by the ongoing task and to prevent deterioration in operational performance, as reflected by increased functional connectivity [12,73]. After fatigue inducement, some brain functions in subjects in the 240 Hz pure tone group were affected, so more regions were activated to work in concert to ensure attention and perceptual effort to maintain the TloadDback performance, and the enhanced functional connectivity in the 240 Hz pure tone group both demonstrated the presence of mental fatigue and explained the stability of their TloadDback performance. In summary, network connectivity analysis showed that the 240 Hz pure tone did not play any role during fatigue inducement. It has been demonstrated that a higher node transmission efficiency and a smaller average path length results in better connectivity and faster information processing in brain networks [73]. Further analysis of topological structure revealed that, after fatigue inducement, the average path length of the 15 Hz binaural beats group significantly decreased and node efficiency showed an upward tendency, demonstrating the effectiveness of the beta frequency band intervention at the neurophysiological level. Binaural beats take advantage of the brain’s structures to actively generate internal oscillatory modulation [53]. Due to the non-linear relationship between binaural beats and cortical activity, there has not been unified opinion on the frequency band of binaural beats that can intervene in fatigue states, but the effectiveness of beta frequency band binaural beats has been confirmed in previous studies. Listening to 15 Hz binaural beats led to improved subject performance in a visual vigilance task and it had an impact on task-related emotional changes [49]. Compared with theta/delta frequency binaural beats, the increase in task-related feelings such as confusion and fatigue were smaller in beta frequency binaural beats interventions. Listening to 15 Hz binaural beats also contributed to improved performance on visuospatial working memory tasks and produced cortical networks with high information transfer characteristics [53,74]. In the present study, 15 Hz binaural beats led to a decrease in average path length and an increasing tendency in node efficiency, and the changes associated with these efficiency gains demonstrate that 15 Hz binaural beats intervention while inducing mental fatigue facilitates the stabilization of TloadDback performance (which contains a working memory task) and produces brain functional networks with high connectivity. Therefore, with the results of this study, we provide new evidence for the effectiveness of 15 Hz binaural beats for mental fatigue intervention that is of great scientific and practical value.

## 5. Limitations

There are some limitations to this study. In our study, we only determined the number of subjects according to the previous literature and did not calculate the sample size in advance. Subsequent research should determine and recruit subjects based on the calculation results of the sample size to strictly control type I and type II errors. Previous studies have shown that, as the number of EEG electrodes increases, the identification and calculation of retrospective and functional brain networks will be more accurate [75,76]. While the electroencephalogram device in this study had 32 electrodes, future studies should use EEG devices with 128 electrodes or more for subsequent precise analysis.

## 6. Conclusions

The present study demonstrated the intervention effect of 15 Hz binaural beats on the enhancement of brain network efficiency and the maintenance of performance in working memory tasks under a mental fatigue state. We suggest that 15 Hz binaural beats may be applied to improve individual performance in areas such as transportation and the medical field, which are limited by the negative effects of mental fatigue. Follow-up studies could be conducted on this group of people using 15 Hz binaural beats to investigate what combination of listening time and number of listens would result in the optimum fatigue intervention, thus resulting in a non-invasive mental fatigue intervention with theoretical and practical value.

## Figures and Tables

**Table 1:** Basic information (M ± SD).

	Age	Scores		
		PSQI	ESS	MES
15 Hz binaural beats group	23.27 ± 3.90	5.13 ± 2.20	5.13 ± 2.50	51.47 ± 8.25
40 Hz binaural beats group	20.80 ± 2.40	4.87 ± 2.45	4.87 ± 3.09	50.73 ± 7.94
relaxing music group	20.47 ± 2.00	3.93 ± 1.28	5.53 ± 2.70	48.93 ± 4.64
240 Hz pure tone group	23.20 ± 3.61	3.40 ± 2.77	4.73 ± 2.94	52.07 ± 2.61

Note: There is no difference between the four groups in the scores of PSQI, ESS, MES, or age.

**Table 2:** TloadDback task results (unit: ms (reaction time); % (correctness)).

	Group	Stage				
		Stage 1	Stage 2	Stage 3	Stage 4	Stage 5
1-back task reaction time	1	579.53 ± 67.68	581.67 ± 52.18	584.16 ± 40.23	571.19 ± 48.16	561.35 ± 52.65
	2	532.78 ± 41.33	534.34 ± 59.17	535.60 ± 58.64	534.44 ± 76.03	523.20 ± 86.75
	3	564.35 ± 90.49	566.19 ±100.64	558.45 ± 116.91	542.80 ± 109.73	541.86 ± 103.16
	4	583.94 ± 97.55	593.53 ± 99.85	583.20 ± 119.32	569.03 ± 123.58	559.99 ± 135.74
odd-even decision task reaction time	1	562.46 ± 58.99	570.77 ± 35.90	573.51 ± 48.99	567.14 ± 57.96	566.34 ± 53.54
	2	535.46 ± 43.81	544.51 ± 51.61	547.42 ± 56.99	548.93 ± 53.77	545.11 ± 58.98
	3	554.59 ± 71.41	556.53 ± 84.34	556.10 ± 98.08	546.38 ± 92.06	550.62 ± 89.08
	4	580.67 ± 98.10	589.39 ± 99.59	588.89 ± 118.34	580.72 ± 123.25	573.47 ± 131.10
accuracy rate	1	0.91 ± 0.04	0.89 ± 0.05	0.89 ± 0.04	0.88 ± 0.05	0.87 ± 0.06
	2	0.87 ± 0.06	0.82 ± 0.07 *	0.82 ± 0.07 *	0.83 ± 0.06 *	0.83 ± 0.08
	3	0.88 ± 0.07	0.86 ± 0.10	0.84 ± 0.09 **	0.85 ± 0.09	0.86 ± 0.08
	4	0.86 ± 0.08	0.85 ± 0.07	0.83 ± 0.07	0.85 ± 0.07	0.83 ± 0.10

Note: * indicates that there is a difference between the accuracy rate on this stage and the first stage, and the significance level is *p* < 0.05; ** indicates that there is a difference between the accuracy rate on this stage and the first stage, and the significance level is *p* < 0.01.

**Table 3:** The topological structure of the theta band in the eyes-opening state.

		Before Fatigue Inducement	After Fatigue Inducement
15 Hz binaural beats group	Clustering coefficient	0.19 ± 0.03	0.19 ± 0.02
	Average path length	1.20 ± 0.13	1.13 ± 0.08 *
	Nodal efficiency	0.17 ± 0.02	0.18 ± 0.01
40 Hz binaural beats group	Clustering coefficient	0.20 ± 0.03	0.20 ± 0.03
	Average path length	1.14 ± 0.16	1.12 ± 0.15
	Nodal efficiency	0.18 ± 0.02	0.18 ± 0.02
Relaxing music group	Clustering factor	0.19 ± 0.04	0.18 ± 0.03
	Average path length	1.21 ± 0.25	1.24 ± 0.27
	Nodal efficiency	0.17 ± 0.03	0.17 ± 0.03
240 Hz pure tone group	Clustering coefficient	0.18 ± 0.03	0.18 ± 0.02
	Average path length	1.19 ± 0.13	1.15 ± 0.08
	Nodal efficiency	0.17 ± 0.02	0.18 ± 0.01

Note: * indicates that there is a difference in that parameter between pre- and post-fatigue inducement, and the significance level is *p* < 0.05.

## Data Availability

The datasets presented in this article are not readily available because the datasets involve unfinished research projects. If necessary, requests to access the datasets should be directed to the corresponding author.
